# FIRST radio galaxy data set containing curated labels of classes FRI, FRII, compact and bent

**DOI:** 10.1016/j.dib.2023.108974

**Published:** 2023-02-11

**Authors:** Florian Griese, Janis Kummer, Patrick L.S. Connor, Marcus Brüggen, Lennart Rustige

**Affiliations:** a Center for Data and Computing in Natural Sciences (CDCS), Notkestrasse 9-11, D-22607 Hamburg, Germany; b Institute for Biomedical Imaging, Hamburg University of Technology, D-21073 Hamburg, Germany; c Section for Biomedical Imaging, University Medical Center Hamburg-Eppendorf, D-20246 Hamburg, Germany; d Universität Hamburg, Hamburger Sternwarte, Gojenbergsweg 112, D-21029 Hamburg, Germany; e Institut für Experimentalphysik, University of Hamburg, Luruper Chaussee 149, D-22761 Germany; f Deutsches Elektronen-Synchrotron DESY, Notkestrasse 85, Hamburg D-22607, Germany

**Keywords:** Radio Galaxy, FIRST survey, FRI, FRII, Bent, Compact, Fanaroff-Riley

## Abstract

Automated classification of astronomical sources is often challenging due to the scarcity of labelled training data. We present a data set with a total number of 2158 data items that contains radio galaxy images with their corresponding morphological labels taken from various catalogues [1,2]. The data set is curated by removing duplicates, ambiguous morphological labels and by different meta data formats. The image data was acquired by the VLA FIRST (Faint Images of the Radio Sky at Twenty-Centimeters) survey [3]. The morphological labels are collected and the catalogue specific classification definition is converted into a 4-class classification scheme: FRI, FRII, Compact and Bent sources. FRI and FRII correspond to the two classes of the widely used Faranoff-Riley classification [4]. We consider two more classes: compact sources and bent-tail galaxies. For duplicates with different morphological labels, the galaxy is regarded as ambiguously labeled and both coordinates are removed. For the remaining list of coordinates, the radio galaxy images are collected from the virtual observatory skyview (https://skyview.gsfc.nasa.gov/current/cgi/query.pl). The gray value images are provided in the size of 300 × 300 pixel and all pixels with a value below three times the local RMS of the noise are set to this threshold value. The data set is useful for the development of robust machine learning models that automate the classification of radio galaxy images.

**Specifications Table**

**Table:** 

Subject	Astronomy and Astrophysics
Specific subject area	Radio astronomy: Morphological Classification of radio galaxy images
Type of data	Image with class label
How data were acquired	Images are collected by coordinate from virtual observatory skyview (https://skyview.gsfc.nasa.gov/current/cgi/query.pl) and labels are collected by coordinate from catalogues
Data format	Raw (png and csv), Filtered (hdf5)
Description of data collection	The radio galaxy images are collected by sending a html request to the virtual observatory skyview (https://skyview.gsfc.nasa.gov/current/cgi/query.pl) with the following parameters: coordinates, sigma =3 and image patch size =300. The website returns gray images with uInt8 data type normalized between 0 and 255. Corresponding to coordinates, the morphological labels are published by the catalogues in supplementary files in form of text files (.txt) or PDFs. Only images from the four classes FRI, FRII, Compact and Bent are considered for the data set.
Data source location	Primary data sources: Radio galaxy images from VLA FIRST (1.4GHz) survey [3]: • Virtual observatory skyview (https://skyview.gsfc.nasa.gov/current/cgi/query.pl) VLA FIRST (1.4 GHz) survey **Provenance**: The FIRST project team: R.J. Becker, D.H. Helfand, R.L. White M.D. Gregg. S.A. Laurent-Muehleisen. **Copyright:** 1994, University of California. Permission is granted for publication and reproduction of this material for scholarly, educational, and private non-commercial use. Inquiries for potential commercial uses should be addressed to: Robert Becker, Physics Dept, University of California, Davis, CA 95616 Institution: NASA City/Town/Region: CA Country: U.S. Latitude and longitude (and GPS coordinates, if possible) for collected samples/data: Label information: • Gendre [5], [6] Catalogue, Table: mnras0404-1719-SD1.pdf, data tables CoNFIG-1 to CoNFIG-4 • Capetti [7], [8], – Catalogue,Table – Catalogue, Table • Baldi [9], Catalogue, Table • Mira Best [10], Catalogue, Table • Proctor [11], Catalogue, Table, data from Table 1 from reference with label “WAT” and “NAT”
Data accessibility	Repository name: RadioGalaxyDataset Data identification number: 10.5281/zenodo.7351724[2] Direct URL to data: https://doi.org/10.5281/zenodo.7351724.
Related research article	J. Kummer, L. Rustige, F. Griese, K. Borras, M. Brüggen, P. L. S. Connor, F. Gaede, G. Kasieczka, P. Schleper, Radio galaxy classification with wgan-supported augmentation, in: INFORMATIK 2022, volume P-326 of Lecture Notes in Informatics (LNI) - Proceedings, Gesellschaft für Informatik, Bonn, 2022, pp. 469-478. doi:10.18420/inf2022_38. [1]

## Value of the Data

• The data set is useful as it provides an easy to access, curated and combined data set based on various catalogues. This data set can be used to develop supervised deep learning models to classify radio galaxies in the categories FRI, FRII, Compact and Bent.
• Computer scientists in the field of Astronomy and Astrophysics who are developing supervised, self-supervised or unsupervised deep learning models for automatic classification, object detection or data generation. Further, the data set can be used to validated and evaluated unsupervised models.
• In combination with a labeled LOFAR data set, the data set can be used to develop a model that generalizes to data from another telescope operating in a different wavelength range.
• The easy-accessible and curated data set is suitable for educational purposes in applying machine learning methods to astronomical data.

## 1 Objective

The data set is created to train supervised deep learning models on radio galaxy data. However, the available data set [12] has a limited number of 1256 data entries. The extraction of data from various catalogues turned out to be challenging because meta data is not consistent. In some catalogues, identical radio galaxy sources have different coordinates due to different rounding schemes. Further, identical radio galaxy sources can have different classification labels between different catalogues. In this data set, data items can easily be filtered by class label, catalogue or coordinate range. Researchers should have the ability to build on this data set and do not have to repeat this work.

## 2 Data Description

**Fig. 1:**
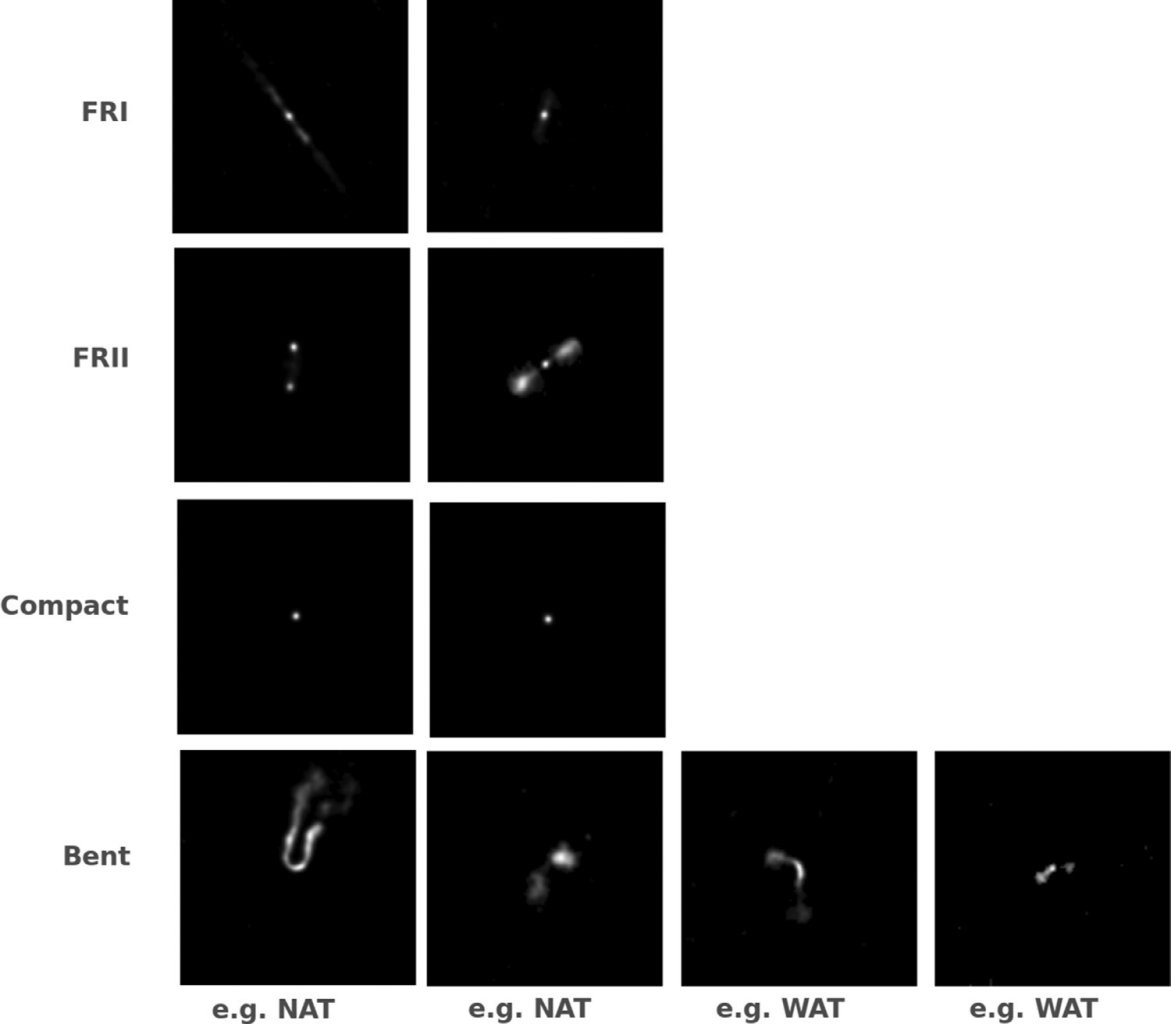
Examples of the classes FRI, FRII, Compact and Bent. The figure is adapted from the original figure in [1].

We combined different catalogues which characterise radio galaxy sources from the FIRST survey [3] to create a data set of radio galaxy images with morphological labels. The labeling is typically done by experts by considering radio images and the corresponding optical counterparts. In this work, we group radio sources in 4 classes (FRI, FRII, Compact and Bent) as done in [13], [14]. FRI and FRII are defined by Fanaroff-Riley in [4]. The Compact class consists of unresolved point sources. The Bent class consists of sources for which the angle between the jets differs significantly from 180 degrees. It contains two subtypes: narrow-angle tail (NAT) and wide-angle tail (WAT) galaxies, depending on the angle between the jets. In Fig. 1 adapted from [1], a few examples of each class are shown. The created data set has a total size of 5.1 MB.

### 2.1 firstgalaxydata/galaxy_data.zip

The data set can be found in the galaxy_data folder by unzipping galaxy_data.zip. It contains the folder structure

• /[all,train,valid,test]/FRI
• /[all,train,valid,test]/FRII
• /[all,train,valid,test]/Compact
• /[all,train,valid,test]/Bent

with corresponding.png images. The most import information will also be part of the file name separated by underscores: ‘RA_DEC_Label_Catalogue.png‘ E.g. ‘14.084_-9.608_3_MiraBest.png‘

The number of radio galaxy sources per class and split are given in Table. 1.

**Table 1:** Number of sources per class in the data set. The table is adapted from the original table in [1].

	FRI	FRII	Compact	Bent	Total (%)
train	395	824	291	248	1758 (81,46%)
validation	50	50	50	50	200 (9,26%)
test	50	50	50	50	200 (9,26%)
total (%)	495 (22,93%)	924 (42,81%)	391 (18,11%)	348 (16,12%)	2158 (100%)

### 2.2 firstgalaxydata/galaxy_data_h5.zip

The combined data set collected from the FIRST catalogues is summarized in the HDF5 file galaxy_data_h5.h5 with a group named ”data_$(i)” for every data entry with i=1,⋯,n with n as the total number of data entries. Each group has the following data sets

• “Img”: two-dimensional uint8 array with (300,300), The data set “Img” has the following attributes
  • “RA”: double, right ascension equatorial coordinate system (J2000)
  • “DEC” double, declination equatorial coordinate system (J2000)
  • “Source”: string [“Gendre”, “MiraBest”, ”Capetti2017a”, ”Capetti2017b”, ”Baldi2018”, ”Proctor_Tab1”]
  • “Filepath_literature”: string, relative path to the *.png file in folder ‘galaxy_data’
• “Label_literature”: double, 0.0: “FRI”, 1.0: “FRII”, 2.0: “Compact”, 3.0: “Bent”
• “Split_literature” : string, [“train”, “valid”, “test”]

### 2.3 firstgalaxydata/firstgalaxydata.py

This python class is able to load the galaxy_data_h5.h5 with several filtering options to provide a data.Dataset class for the pytorch framework.

### 2.4 firstgalaxydata/Example_firstgalaxydata.py

This file shows example code on how to use the firstgalaxydata.py class.

### 2.5 requirements.txt

The requirements.txt contains the necessary packages in order to use the firstgalaxydata.py class with python.

### 2.6 meta/FRICat_Capetti_2017_relabeled.csv

The FRICat_Capetti_2017_relabeled.csv contains the relabeled sources from FRICat Capetti catalogue as shown in Table. 2.

**Table 2:** Re-labeled galaxies from FRICat Capetti catalogue [7].

	RA in ${}^{\circ }$	DEC in ${}^{\circ }$	Original Label	Corrected Label
1	140.204	40.665	FRI	Bent
2	152.017	50.445	FRI	Bent
3	159.613	41.815	FRI	Bent
4	181.006	20.232	FRI	Bent
5	181.105	3.753	FRI	Bent
6	216.568	0.838	FRI	Bent
7	223.064	50.374	FRI	Bent
8	227.489	33.454	FRI	Bent
9	250.225	32.791	FRI	Bent

### 2.7 meta/galaxy_data_removed.csv

The galaxy_data_removed.csv file contains a list of sources that are not included in the data set. These sources are already added to the data set but have slightly different coordinates within the deviation of $\pm 0.015^{\circ }$ in right ascension (RA) and declination (DEC). Sources with different coordinates within the deviation are regarded as duplicates.

### 2.8 meta/galaxy_data_different_labels.csv

The galaxy_data_different_labels.csv contains a list of pairs of coordinates which are within the deviation of $\pm 0.015^{\circ }$ in right ascension (RA) and declination (DEC) but in this case the label information is different from the original catalogues. These source coordinates are entirely excluded from the data set.

### 2.9 img/Classification_Scheme.png

An image showning examples of the four classes.

## 3 Experimental Design, Materials and Methods

The morphological labels are collected and assigned to the corresponding class from the following catalogues. For mapping the labels to the radio galaxy images, the equatorial coordinates (J2000) with right ascension (RA) and declination (DEC) are used. For the FRI class, we used the catalogue of [5], [6] by collecting images from data tables CoNFIG-1 to CoNFIG-4 with label “I”. Additionaly, images from the catalogue of [10] were collected with label “0” and the images from the catalogue of [7] were selected. For the FRII class, we used the catalogue of [5], [6] by collecting images from data tables CoNFIG-1 to CoNFIG-4 with label “II” and “IIc”. The catalogue of [10] with images with label “5” and “6” provided further images of FRII along with the images of the catalogue of [8]. For the Compact class, we used the catalogue of [5], [6] by collecting images from data tables CoNFIG-1 to CoNFIG-4 with label “C”, “C*” and “S*”. Further, the catalogue of [9] was used for Compact. For the Bent class, we used the catalogue of [5], [6] by collecting images from data tables CoNFIG-1 to CoNFIG-4 with label “Iw”. Further, we selected bent-type sources from [11] by collecting only from Table 1 from [11] with label “WAT” and “NAT”. From catalogue [10] we collected images with label “1” and “2” for the Bent class.

We identified 300 source coordinates within a deviation of $\pm 0.015^{\circ }$ in right ascension (RA) and declination (DEC) from different catalogues. 227 of 300 source coordinates had the same label information from different catalogues. Here, the radio galaxy image is only added once to data set and the listed 227 source coordinates are regarded as duplicates. 73 of 300 source coordinates had different label information from different catalogues. Here, the radio galaxy images are ambiguously labeled and excluded from the data set entirely. From the FRICAT catalogue [7] 9 sources were manually re-labeled from FRI to Bent since these sources are NAT galaxies. The coordinates of the re-labeled galaxies are listed in Table. 2.

### 3.1 Preprocessing

We downloaded the images of the FIRST survey from the virtual observatory skyview (https://skyview.gsfc.nasa.gov) using the equatorial coordinates (J2000). The original images size is 300 x 300 pixel. We adopted the preprocessing and the choice of preprocessing parameters from [15] and [13]. At first, all NaN value are set to zero. Second, all pixel values below three times the local RMS noise are set to the value of this threshold with help of the following functions sigma_clipped_stats and preprocess_clip_normalize. The value of sigma equal to 3 ensures that the background noise of the images is cleared. We have used python version 3.6.8, numpy version 1.19.5 and astropy version 4.1.



## Ethics Statements

The authors comply with redistribution polices of the primary data sources and have stated the license for the reuse of primary data.

## CRediT authorship contribution statement

**Florian Griese:** Conceptualization, Methodology, Data curation, Software, Writing – original draft. **Janis Kummer:** Conceptualization, Data curation, Validation, Writing – review & editing. **Patrick L.S. Connor:** Writing – review & editing. **Marcus Brüggen:** Writing – review & editing, Supervision, Funding acquisition. **Lennart Rustige:** Data curation, Validation, Writing – review & editing.

## Declaration of Competing Interest

The authors declare that they have no known competing financial interests or personal relationships which have, or could be perceived to have, influenced the work reported in this article.

## Data Availability

RadioGalaxyDataset (Original data) (Zenodo).
